# Supplementary material for: Rare and Common Variants Uncover the Role of the Atria in Coarctation of the Aorta
Source: Genes (Basel). 2022 Apr 2;13(4):636. doi: 10.3390/genes13040636 (PMC9032275; doi:10.3390/genes13040636)
Supplement: Supplementary file 1 [file genes-13-00636-s001.zip › genes-1623476-supplementary.pdf]

Supplementary materials

Supplementary Figure S1. Bioinformatics pipeline

Supplementary Table S1. Rare *MYH6* damaging variants in Pittsburgh LVOTO subjects.

Supplementary Table S2. Significant excess of *MYH6* rare variants in CoA in Pittsburgh LVOTO subjects.

Supplementary Table S3. Summary of genetic evidence of *MYH6* to different LVOTO lesions.

Supplementary Table S4. Prevalence of *PCDHA* delCNV and *MYH6* damaging variants in Pittsburgh LVOTO subjects.

Supplementary Table S5. Analysis for co-occurrence of *PCDHA* delCNV and rare damaging *MYH6* variants.

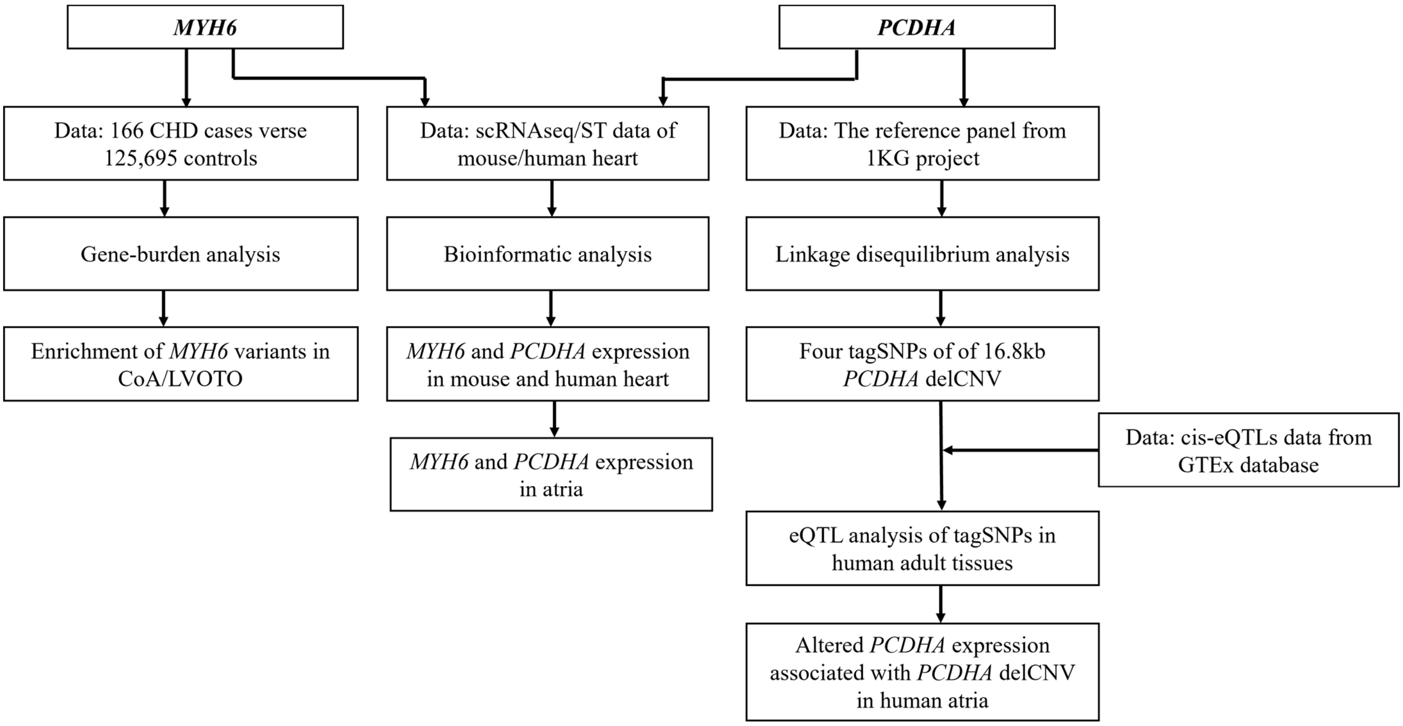

Supplementary Figure S1. Bioinformatics pipeline

scRNAseq: single cell RNA sequencing. ST: spatial transcriptomics. 1KG: 1000 genome project. eQTL: expression Quantitative Trait Loci. GTEx: The Genotype-Tissue Expression.

Supplementary Table S1. Rare *MYH6* damaging variants in Pittsburgh LVOTO subjects

Sample ID	Sex	*PCDHA* delCNV	Chr:Position	*MYH6 variants*									CHD Phenotype				
				Ref	Alt	dbSNP150	Allele Depth	Genotype	CADD	MAF	Variant	Variant Classification	BAV	CoA	HLHS	Aortic arch hypoplasia/Interrupted aortic arch	Phenotype Catogary
7009	M	+/Δ16kb	14:23868213	A	ACTC	--	83,68	0/1	--	--	c.1614_1615insGAG: p.C539delinsEC	nonframeshift insertion		✓		✓	Isolated_CoA
7010	F	+/+	14:23863081	C	T	rs758061689	83,84	0/1	26.5	3.18E-05	c.G2722A:p.D908N	missense		✓			Complex_CoA
7164	M	+/+	14:23863081	C	T	rs758061689	95,93	0/1	26.5	3.18E-05	c.G2722A:p.D908N	missense	✓				Isolated_BAV
7289	M	+/Δ16kb	14:23858207	G	A	rs372736126	6,5	0/1	34	1.60E-05	c.C4036T:p.R1346W	missense			✓		HLHS
7340	M	+/+	14:23866178	C	T	rs770907731	30,21	0/1	26.8	.	c.G2162A:p.R721Q**	missense	✓	✓			BAV/CoA
7340	M	+/+	14:23854157	C	T	rs142437308	10,27	0/1	25	3.98E-06	c.G5257A:p.A1753T	missense	✓	✓			BAV/CoA
7494	M	+/+	14:23857395	G	T	rs727503234	112,80	0/1	31	8.35E-05	c.C4328A:p.A1443D	missense	✓	✓			BAV/CoA
7532	M	+/+	14:23855280	C	T	rs534560839	57,58	0/1	25.1	0.0001	c.G5020A:p.A1674T	missense			✓		HLHS
7537	F	+/+	14:23868075	C	T	rs150415679	120,107	0/1	28.2	0.0001	c.G1753A:p.G585S	missense		✓		✓	Complex_CoA
7575	F	+/+	14:23871741	A	G	--	57,52	0/1	23.1	.	c.T1073C:p.M358T	missense	✓	✓			BAV/CoA
7711	M	+/+	14:23865584	C	T	rs748960382	30,41	0/1	29	3.98E-06	c.G2338A:p.D780N	missense	✓	✓		✓	BAV/CoA

**This amino acid substitution significantly associated with CoA in Iceland population (Bjornsson et al. 2018).

Minor allele frequency of these variants should be less than 0.0002 in GnomAD exome v2.1.1.

Supplementary Table S2. Significant excess of *MYH6* rare variants in CoA within the Pittsburgh LVOTO

	MYH6 carriers	non-*MYH6* carriers	Frequency %	*P*	OR (CI 95)
non-CoA LVOTO (n=111)	3	108	2.7	0.01601	5.19 (1.13-32.44)
CoA (n=55)	7	48	12.7		

Fisher's exact test was used to estimate P value, odds ratio (OR) and 95% confidence interval (95%CI).

Supplementary Table S3. Analysis of the association of *MYH6* variants to different LVOTO lesions

a. Using GnomAD Exome v2.1.1 as Reference Population

Cohort (sample size)	No. of *MYH6* carriers	Frequency (%)	*P*	OR (95%CI)
Reference population: GnomAD exome v2.1.1, n=125,695	975	0.776		
*LVOTO (166)	10	6.02	**9.12e-07**	**8.20 (3.84-15.55)**
Isolated BAV (37)	1	2.78		ns
HLHS (58)	2	3.45		ns
non-CoA LVOTO (n=111)	3	2.7		ns
*All CoA (55)	7	12.73	**2.54e-07**	**18.64 (7.10-41.59)**
*BAV/CoA (29)	4	13.79	**7.43e-05**	**20.47 (5.17-59.44)**
*Complex CoA (15)	2	13.33	**0.005924**	**19.67 (2.15-87.28)**
Isolated CoA (11)	1	9.09		ns

b. Using GnomAD Exome v2.1.1 non-Finnish European as Control Reference Population

Cohort (sample size)	No. of *MYH6* carriers	Frequency (%)	*P*	OR (95%CI)
Reference population: GnomAD exome v2.1.1 non-Finnish European, n=56,853	543	0.955		
*LVOTO (166)	10	6.02	**5.85e-06**	**6.65 (3.11-12.64)**
Isolated BAV (37)	1	2.78		ns
HLHS (58)	2	3.45		ns
non-CoA LVOTO (n=111)	3	2.7		ns
*All CoA (55)	7	12.73	**1.03e-06**	**15.12 (5.75-33.81)**
*BAV/CoA (29)	4	13.79	**1.66e-04**	**16.59 (4.18-48.28)**
Complex CoA (15)	2	13.33	0.008863	15.95 (1.74-70.75)
Isolated CoA (11)	1	9.09		ns

Summary of *MYH6* rare variant frequency in LVOTO cohorts and reference population (GnomAD exome v2.1.1, all samples or non-Finnish European samples). Fisher's exact test was used to estimate *P* value, odds ratio (OR) and 95% confidence interval (95%CI) in analysis for excess *MYH6* rare variants in LVOTO cohort vs. reference population (ns=not significant if $P>0.05$). *Statically significant Fisher’s exact test ($P<0.00714$ correction for multiple testing threshold). All LVOTO subjects in this study are European population. Reference cohort is GnomAD exome v2.1.1.

Supplementary Table S4. Prevalence of *PCDHA* delCNV and *MYH6* damaging variants in Pittsburgh LVOTO subjects

Phenotype	No. Subjects	*PCDHA delCNV* only	*MYH6* only	*PCHDA delCNV* and *MYH6*	TOTAL
All LVOTO	166	34 (20.48%)	8 (4.82%)	2 (1.21%)	44 (26.5%)
Isolated BAV	37	8 (21.62%)	1 (2.70%)	0 (0.00%)	9 (24.32%)
Complex BAV	7	3 (42.86%)	0 (0.00%)	0 (0.00%)	3 (42.86%)
HLHS	58	9 (15.52%)	1 (1.72%)	1 (1.72%)	11 (18.97%)
All CoA	55	11 (20.00%)	6 (10.90%)	1 (1.82%)	18 (32.73%)
Isolated CoA	11	4 (36.36%)	0 (0.00%)	1 (9.09%)	5 (45.45%)
Complex CoA	15	3 (20.00%)	2 (13.33%)	0(0.00%)	5 (33.33%)
BAV/CoA	29	4 (13.79%)	4 (13.79%)	0 (0.00%)	8 (27.59%)

Supplementary Table S5. Analysis for co-occurrence of *PCDHA* delCNV and rare damaging *MYH6* variants

	***MYH6* and *PCDHA* delCNV**	***MYH6* or *PCDHA* delCNV**	***P*-value**
LVOTO (n=44)	2 (4.3%)	42 (95.7%)	0.66
Controls * (n=68)	2 (2.9%)	66 (97.1%)	

Due to higher variant load in LVOTO patients, the analysis was limited to only individuals with at least one allele of *MYH6* or *PCDHA* delCNV. Individuals with neither variant were excluded.

Fisher's exact test was used to estimate *P* value.

*Controls are from 1KG Non-Finnish European population.
